# Garlic, Onion, and Cinnamon Essential Oil Anti-Biofilms’ Effect against *Listeria monocytogenes*

**DOI:** 10.3390/foods9050567

**Published:** 2020-05-04

**Authors:** Mariem Somrani, María-Carmen Inglés, Hajer Debbabi, Ferid Abidi, Alfredo Palop

**Affiliations:** 1 Departamento de Ingeniería Agronómica, Instituto de Biotecnología Vegetal, Universidad Politécnica de Cartagena, 30202 Cartagena, Spain; mari.somrani@gmail.com; 2 Department of AgriFood Industries, UR17AGR01-PATIO, National Agronomic Institute of Tunisia, University of Carthage, 1082 Tunis, Tunisia; debbabih@gmail.com; 3 Scentium Flavours, 30840 Alhama de Murcia, Spain; mcingles@scentium.com; 4 Laboratory of Protein Engineering and Bioactive Molecules (LIP-MB), National Institute of Applied Sciences and Technology, University of Carthage, 1080 Tunis, Tunisia; feridinsat@yahoo.fr

**Keywords:** essential oil, garlic, onion, cinnamon, biofilm, *Listeria monocytogenes*

## Abstract

Biofilms represent a serious problem for food industries due to their persistence in processing surfaces, from which they can cause food spoilage or, even worse, lead to foodborne diseases. Microorganisms immersed in biofilms are more resistant to biocides. The search for natural effective alternatives for the prevention and the control of biofilms has increased lately. The aim of this research was to test the antibacterial and the anti-biofilm activities of cinnamon, onion, and garlic essential oils against *Listeria monocytogenes*. The methodology highlighted first the effect of these essential oils on *L. monocytogenes* using disc diffusion and minimum inhibitory concentration (MIC) methods and then on initial cell attachment and six hours preformed biofilms. The inhibition of biofilms was assessed by crystal violet assay. Sulfides were the most abundant compounds present in onion and garlic essential oils, while cinnamaldehyde was predominant in cinnamon essential oil. MIC values were of 0.025 mg mL^−1^ for onion essential oil and 0.100 mg mL^−1^ for cinnamon and garlic. Onion essential oil inhibited initial cell attachment by 77% at 0.5 of the MIC dose, while at MIC, cinnamon and garlic essential oils inhibited the initial microbial adhesion completely. All three essential oils completely inhibited initial cell attachment when applied at 2 MIC. On the contrary, preformed biofilms were more resistant, and the inhibition rate ranged from 33% to 78%. In summary, this investigation revealed that the essential oils of garlic, onion, and cinnamon show an effective antibiofilm activity against *L. monocytogenes* and are promising natural antimicrobial alternatives for food processing facilities.

## 1. Introduction

*Listeria monocytogenes* is a Gram-positive bacterium that is commonly present in the environment as well as in food processing facilities, especially in inaccessible parts of industrial equipment and materials, where it can last up to 10 years [1]. It is a foodborne pathogen. Listeriosis is a disease that predominantly affects immunocompromised persons, such as the elderly, immunosuppressed people, and pregnant women, together with their unborn or new-born babies. It is of particular concern because of its high mortality rate. For example, it caused the highest number of food-borne disease-related deaths in Europe by 2018 [2]. The hazard of *L. monocytogenes* consists, not only in its ability to grow under refrigeration temperatures but also in its ability to form biofilms, which are difficult to eliminate during the cleaning process [3,4,5].

Biofilms are an assemblage of microorganisms in which microbial cells are embedded in a self-produced matrix of an extracellular polymeric substance, mainly composed of polysaccharides [6]. They are one of the most serious problems in the food industry due to their formation and persistence on processing surfaces, which can cause food spoilage and contamination and, if the biofilm is composed of foodborne pathogen bacteria, food diseases [7]. Bacteria present in a biofilm enjoy additional advantages, including enhanced survival to stressing conditions, such as high temperatures, ultraviolet rays, and biocides [8]. In general, biocides are more effective against planktonic microorganisms than when these microorganisms are immersed in a biofilm. The complex and compact structure within biofilms makes it difficult for biocides to penetrate and reach internal layers [9,10]. Moreover, most biocides are not eco-friendly and may pose health risks to humans [11].

Thus, the study of new tools for the control of bacterial biofilms has increased lately, focusing on novel technologies, such as cold atmospheric plasma [12], or natural antimicrobials. Among these natural antimicrobials, essential oils (EOs) have always been among the best options to choose as they come from edible plants that have been consumed by humanity since ancient times. As a consequence, they have been generally recognized as safe (GRAS) by the Food and Drug Administration [13]. An essential oil is a concentrated, volatile, and aromatic liquid, which can be obtained from different parts of plant material [14]. *Cinnamomum cassia* is among the plants that are sources of essential oils with antioxidant and antimicrobial properties against foodborne pathogen and spoilage microorganisms. Its composition, rich in terpenes and other aromatic compounds, is behind these properties [15,16]. In addition, *Allium sativum* and *Allium cepa* EOs are known for their antimicrobial effect due to the powerful sulfur and other numerous phenolic compounds [17]. Besides, different studies have been published on the antimicrobial activities of plant compounds against *L. monocytogenes* [18,19,20,21,22]. However, their antibiofilm effects are much less known. Therefore, the aim of this study was to investigate the effect of three EOs (onion, garlic, and cinnamon) on *L. monocytogenes* biofilms.

## 2. Materials and Methods

### 2.1. Bacterial Culture

*L. monocytogenes* CECT 4032 was used in this study, and it was provided by the Spanish Type Culture Collection (CECT, Valencia, Spain). This strain was stored at 80 °C (30% glycerol) until use. For the experiments, a fresh culture of *L. monocytogenes* was subcultured on Brain Heart Infusion Agar (BHI, Scharlau Chemie, Barcelona, Spain) and incubated at 37 °C for 24 h. Prior to each experiment, a loopful of the BHI culture was inoculated in Tryptone Soya Broth (TSB, Scharlau) and incubated at 37 °C for 24 h.

### 2.2. Essential Oils

The EOs of onion (*A. cepa*, EOO), garlic (*A. sativum*, EOG), and cinnamon (*C. cassia*, EOC) were obtained from Iberchem (Murcia, Spain). Stock solutions were prepared in 95% (v/v) ethanol and stored at 4 °C until used.

### 2.3. Identification of the Essential Oil Components

Identification of the essential oil volatile components was carried out with a gas chromatography-mass spectrometry (GC-MS) system (7890B GC/5977AMSD; Agilent Technologies Inc., Santa Clara, CA, USA), fitted with a 30 m × 0.25 mm × 0.25 µm HP-5 column (Agilent). Samples of 0.3 µL were injected in the split mode at a ratio of 1:150. The oven temperature was programmed as follows: 65 °C hold for seven min, then 5 °C/min to 200 °C, hold for 10 min, and finally, 30 °C/min to 250 °C and hold for 10 min. Helium was used as carrier gas at a flow rate of 1.17 mL/min. The injector and transfer line temperatures were 250 °C and 280 °C, respectively. Mass spectra matches were made by comparing experimental mass spectra with those of the NIST14 computer library.

Semi-quantification of the volatile substances was performed using gas chromatography coupled to a flame ionization detector (6890 N, Agilent) fitted with the same column and operated under the same conditions as the GC-MS. Quantification was computed as the percentage contribution of each compound to the total amount present. The analysis was repeated twice for each sample.

### 2.4. Agar Disk Diffusion Assay

The disc diffusion method was implemented as described by The European Committee on Antimicrobial Susceptibility Testing [23] with some modifications, to test the susceptibility of *L. monocytogenes* to the EOs. The test was performed by applying a 100 µL bacterial inoculum of approximately 1–2 × 10^8^ CFU mL^−1^ to the surface of a Mueller–Hinton agar plate (M.H, Scharlab, Barcelona, Spain). The inoculum was allowed to dry for 15 min, then three sterile disks of 6 mm (BDsensi-disc, Becton Dickinson GMBH, Heidelberg, Germany) were placed on the inoculated agar surface. One was impregnated with 10 µL of one of the EOs, which represents a concentration of 50 mg mL^−1^, another with 10 µL of ethanol, which represented the negative control, and the last one with erythromycin (Duchefa Biochemie, Haarlem, The Netherlands), which was dissolved in 95% ethanol and then added (15 µg/disc) and represented the positive control. The plates were left 15 min at room temperature to allow the diffusion of the compounds, and then they were incubated for 24 h at 35 ± 1 °C. After incubation, the zones of growth inhibition around each of the disks were measured in millimeters. The diameter of the zone was related to the susceptibility of the strain to the EO. The experiments were carried out in triplicate.

### 2.5. Determination of Minimal Inhibitory Concentration (MIC)

One of the antimicrobial susceptibility testing methods was the macrobroth or tube-dilution method that was done, as described by Balouiri et al. [24]. This procedure involved preparing two-fold dilutions of essential oils ranging from 0.2 mg mL^−1^ until 0.00625 mg mL^−1^ in 5 mL of TSB dispensed in test tubes. The EOs-containing tubes were inoculated with a 0.1 mL standardized bacterial suspension of 10^6^ colony forming units (CFU) mL^−1^, prepared from the 24 h at 35 ± 1 °C culture in TSB by appropriate dilution in sterile TSB. After 18 h incubation, the tubes were examined for visible bacterial growth, as evidenced by turbidity. The lowest concentration of EO that prevented visible growth represented the minimal inhibitory concentration (MIC). The experiments were carried out in triplicate.

### 2.6. Inhibition of Initial Cell Attachment

An experiment was performed to evaluate the effect of the different EOs on biofilm formation and specifically on initial cell attachment, as described by Sandasi et al. [25] with few modifications. Solutions of antimicrobials (equivalent to 0.5 MIC, 1 MIC, and 2 MIC) were prepared. One hundred microliters of each solution was added to the flat-bottom 96-well Polystyrene microtiter plates, and equal volumes of ethanol and erythromycin were added as negative and positive controls, respectively. Two hundred microliters of the standardized culture of 10^6^ CFU mL^−1^ (prepared as described above) was added into each well. Equal volumes of ethanol were added as negative controls, while erythromycin was added as a positive control. Wells with only the culture and with uninoculated TSB were also prepared as additional growth and sterility controls to ensure the growth of the microorganism and the sterility of the medium during the experiment. The samples were prepared in triplicate. The plates were sterile sealed and incubated at 37 °C for 24 h to allow cell attachment and biofilm formation. Biofilm biomass was determined, after incubation, using the modified crystal violet assay as described below. The results were expressed as percentage of inhibition (Equation (1)):

*PI* = [(*OD_gc_* − *OD_exp_*)/*OD_gc_*] × 100, (1)

where *PI* is the percentage of inhibition, *OD_gc_* is the optical density of the growth control culture measured at 595 nm, and *OD_exp_* represents the optical density of the cultures incubated with different EOs.

### 2.7. Inhibition of Preformed Biofilm

This experiment was performed as described by Sandasi et al. [26] with a few modifications. Biofilms were allowed to be formed by adding 100 μL of the standardized culture of the 10^6^ UFC mL^−1^ (prepared as described above) into selected wells of the microtiter plate in triplicate and incubating them at 35 ± 1 °C for six hours, allowing the cells to be attached before adding the EOs. After six hours, 100 μL of each stock solution of the EOs was added to each well to achieve a final volume of 200 μL. Equal volumes of ethanol were added as negative controls, while erythromycin was added as a positive control. Wells with only the culture and with uninoculated TSB were also prepared as additional growth and sterility controls to ensure the growth of the microorganism and the sterility of the medium during the experiment. After the treatment of preformed biofilms with antimicrobials, the plates were incubated over a series of time intervals (1, 5, and 20 h) to characterize the effect of the antimicrobial at every defined time. At each time interval, the biofilm biomass was measured using the crystal violet assay, as described below. The results were expressed as percentage inhibition (Equation (1)).

### 2.8. Biofilm Biomass (Crystal Violet Assay)

This method was adopted from Djordjevic et al. [27] with some modifications. For all initial cell attachment and preformed biofilms, the culture medium from each well was gently removed, and the microtiter plates were washed five times with sterile distilled water to remove planktonic bacteria. Then, the plates were air-dried for 45 min. Afterward, 100 µL of 1% crystal violet was added to each well to stain the adhered bacteria, and the plates were stored at room temperature for an additional 45 min. Each microtiter plate was then washed five times with distilled water, and as much liquid as possible was disposed to remove any crystal violet that was not specifically staining the adhered bacteria. One hundred and twenty-five microliters of 95% ethanol was then added to the wells, and they were incubated for 15 min at room temperature to solubilize the remaining crystal violet. One hundred microliters of the destaining solution from each well was then transferred to a new plate, and the optical density was measured at 595 nm (OD_595 nm_) using a microplate reader (Bioscreen C analyzer, Lab Systems, Helsinki, Finland). The OD_595 nm_ was used for determining the percentage inhibition of biomass formation for each concentration of antimicrobial according to Equation (1).

Inhibition was plotted against the extract concentrations using Microsoft Excel^®^.

### 2.9. Statistical Analysis of Quantitative Data

For each quantitative assay, the values obtained were tested using Analysis of Variance (ANOVA) with Microsoft Excel to examine any significant differences. Post-hoc least significant difference (LSD) pairwise multiple comparison tests were then used to look for significant differences between groups. *p* < 0.05 was considered statistically significant.

## 3. Results

### 3.1. Chemical Composition of the Essential Oils

The chemical composition of the EO of garlic, onion and cinnamon is shown in Table 1. The major compounds identified by GC-MS in EOG were diallyl trisulfide (25.13%) and diallyl disulfide (22.74%). Trisulfide dipropyl (35.46%) and dipropyl disulfide (31.11%) were the main compounds of EOO. All they are organo-sulfur compounds regarded as thiosulfates. EOC was mainly composed of E-cinnamaldehyde (76.54%), which belongs to the family of phenylpropanoids.

### 3.2. Antimicrobial Activity

The antimicrobial activities of EOO, EOG, and EOC were assessed by the size of the inhibition zones in the disc diffusion assays and by the MIC values. As shown in Table 2, all EOs exhibited an antimicrobial activity against *L. monocytogenes*, EOO being the essential oil that showed the strongest inhibition zone (37.8 ± 0.4 mm) and EOC the weakest (12.3 ± 0.5 mm), while EOG was in between both values (31.0 ± 1.7 mm). Significant differences (*p* < 0.05) between the three EOs were shown.

Regarding the MIC values, EOG and EOC exhibited the same value (0.1 mg mL^−1^), while EOO showed the lowest value (0.025 mg mL^−1^), i.e., the highest inhibitory effect (Table 2), in accordance to the previous result on the inhibition zones.

### 3.3. Inhibition of Initial Cell Attachment

**Figure 1:**
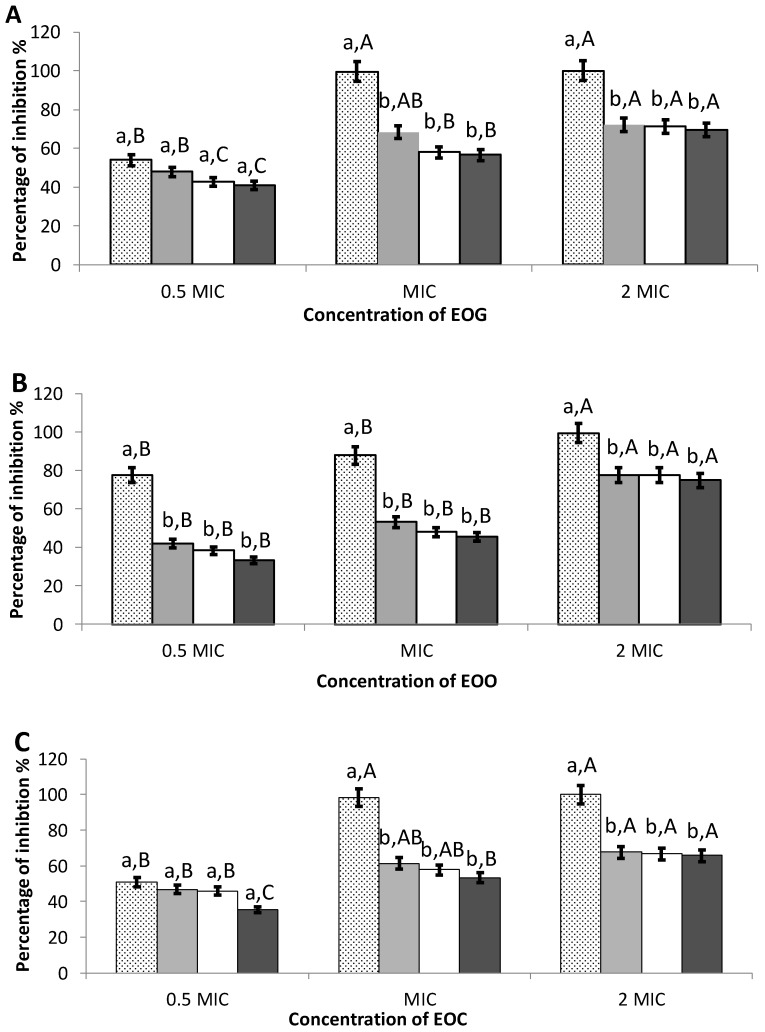
Effect of different concentrations of essential oil of garlic (**A**), onion (**B**), and cinnamon (**C**) on initial cell attachment (dotted column) and preformed biofilms of *L. monocytogenes* incubated with the oil for 1 h (grey columns), 5 h (white columns), and 20 h (black columns). MIC: Minimum Inhibitory Concentration (mg ml^−1^). Error bars indicate standard deviation. a–b, different lower-case letters mean significant differences between different times of incubation with the oil within the same concentration. A–C, different upper-case letters mean significant differences between different concentrations of oil within the same time of incubation (*p* < 0.05).

The percentage of inhibition of cell attachment was high for the three EOs, as shown in Figure 1. Even using the 0.5 MIC dose, EOC and EOG inhibited cell attachment by about 50%, and EOO showed a higher anti-biofilm effect, reaching 77% inhibition. The MIC of EOC and EOG was able to inhibit cell attachment of *L. monocytogenes* completely. Surprisingly, the MIC of EOO did not reach complete inhibition when it was the most inhibiting essential oil at 0.5 MIC and the one that showed the highest inhibitory effect in the in vitro antimicrobial assays. Still, the level of inhibition reached was 88%, quite close to full inhibition. For the 2 MIC value, the three EOs inhibited at 100% the initial cell attachment. The inhibition effect of EOO was dosage-dependent (*p* < 0.05). 

### 3.4. Preformed Biofilms

EOs could also be used to work out a surface treatment on preformed biofilm in the food industry to eliminate contamination. The inhibition of preformed biofilms was 61% for EOC, 53% for EOO, and 68% for EOG after 1 h incubation with the MIC (Figure 1). Again, the higher was the dose (from 0.5 MIC to 2 MIC), the more efficient was the effect of the EO in all three cases (*p*< 0.05). Still, the percentages of inhibition were much lower for preformed biofilms than for the initial cell attachment, in all cases except for 0.5 MIC EOG and EOC, in which they were similar. In 0.5 MIC EOG and EOC, the percentage of inhibition was also rather low for initial cell attachment.

At 0.5 and 1 MIC, a slight decrease in the percentage of inhibition along with incubation time was observed for all three EOs. This decrease was not observed at 2 MIC for any of the EOs.

## 4. Discussion

The chemical composition of the EO of garlic, onion and cinnamon used in this research is somehow similar to what it has been reported in previous researches for these EOs. El-Sayed et al. [28] and Zhang et al. [29] found the same major compounds when analyzing their EOG than the ones found in our work, although the percentages they found were different in each case. This variation could be related to the effects of genetic diversity, geographical origin, harvest factors, or even to the extraction procedures used [30]. Concerning the EOC, Sun et al. [31] obtained in their analysis a percentage of cinnamaldehyde close to our value (79.39%), but Zhang et al. [32] reported that this percentage was as high as 92.40%. They also stated that the amount of a compound depends on the place where the plant has been harvested and on the parts of the plant used to obtain the EO.

Our results showed that all three EOs inhibited the growth of our strain of *L. monocytogenes*, EOO being the one having the strongest effect. Vazquez-Armenta et al. [33] evaluated the effects of EOO on microbial growth and reported the same results concerning the main constituents identified, which, such as sulfur compounds, are responsible for the inhibition of the bacterial key enzymes activity. It has been reported [28] that these sulfur compounds react with the sulfhydryl (SH) groups of cellular proteins, generating mixed disulfides able to damage the microbial cells.

Rohani et al. [19] reported results similar to ours regarding the MIC value of EOG, which was 100 µg mL^−1^ for *L. monocytogenes* ATCC 19118. The data obtained in this work about the antimicrobial activity of EOG could be explained, as for EOO, because of the presence of predominant sulfur compounds. Besides the importance of the number of disulfide bonds, the presence of the allyl group(s) in the EOG is also fundamental for manifesting its antibacterial activity [28].

For EOC, the MIC reported by Mith et al. [34] for *L. monocytogenes* strain NCTC 11,994 was 0.5 µL mL^−1^, while *L. monocytogenes* strain S0580 had a MIC value of 0.25µL mL^−1^ for both *C. cassia* and *Cinnamomum verum* EOs. Hence, there was a variation in sensitivity to EOs between *Cinnamomum* species and also between *Listeria* strains. The different MICs found in our research for this EO could be related to the different strain of *Listeria* used in our case.

The presence of cinnamaldehyde as the most abundant compound in EOC and its broad-spectrum antimicrobial activities were also reported by Bouhdid et al. [35]. EOC antimicrobial activities have been related to its capacity to affect the cell envelope structure, destroying the cytoplasmic membrane [36].

Although EOC showed a lower inhibition zone in the disc diffusion assay compared to EOG, they presented the same MIC value. Golus et al. [37] explained this as a variation related to the nature of the medium, changing from liquid to agar because of water-insoluble compounds’ dispersal in the liquid growth medium. Despite the use of emulsifying agents, the hydrophobic oily substances are often poorly soluble in the liquid medium, and separation of the oil–water phases occurs. Furthermore, this difference could be due to the difficulty of diffusion in agar medium or to the evaporation of volatile components of EOs during the incubation time.

The differences between the three EOs shown in the initial cell attachment could be explained in the differences in their composition (Table 1). These different compositions also explain their antimicrobial effects. The inhibition of initial biofilm formation has already been demonstrated on *L. monocytogenes* and other pathogens for some EOs and their phenolic compounds. Jadhav et al. [38], as well as Sandasi et al. [26], reported results similar to ours, stating that the activity of natural antimicrobials, such as yarrow EO, selected culinary herbs, and medicinal plants, was effective in reducing the initial cell attachment of *L. monocytogenes*. Mohammadi-Bazargani and Rohloff [39] investigated the activity of coriander EO, hexane extract of anise, and methanol extract of peppermint and found that they could inhibit bacterial adhesion of *Staphylococcus aureus* completely. These same authors [39] showed a strong anti-adhesion activity of peppermint oil against *Escherichia coli* with an inhibition value of 98.4%. These results were also consistent with the findings of Lang et al. [40], who reported the effectiveness of a blend at 0.5% concentration of *C. cassia, A. sativum*, and *Mentha piperita* EOs on early biofilm development stages of *Pseudomonas aeruginosa* by preventing the initial attachment of the cells to the surface. Kačániová et al. [41] also found that coriander EO was effective for the inhibition of biofilm formation in microorganisms, such as *Stenotropomonas maltophilia*, *Bacillus subtilis*, and *Penicillium expansum*. Since biofilms are difficult to control due to the enhanced resistance of the bacteria within, preventive inhibition of their formation seems a promising approach. This can be achieved by inhibiting the initial colonization step of the biofilm lifecycle [42].

The three EOs have been much more effective on initial cell attachment than on preformed biofilms, which confirms that bacteria cells within the biofilms develop more resistance to antimicrobials than planktonic cells. Therefore, they are more difficult to eliminate [43]. The biofilm resistance over their planktonic counterparts may be related to the exopolysaccharide matrix, which can act as an impermeable barrier that protects the cells to prevent antimicrobial penetration. Albano et al. [44] showed that the thickness of *Staph. epidermidis* biofilm decreased significantly in the presence of cinnamaldehyde, with a notable alteration in the architecture as the antimicrobial passed through the polysaccharide matrix of the biofilm and caused its perturbation. It may also be related to the biofilm’s high population density, as the cells within being physiologically different from planktonic ones and express specific protection factors [45]. Moreover, the frequency of mutations in bacteria growing in biofilms is significantly higher in comparison to bacteria growing in the planktonic environment, which enhances drug resistance of sessile cells [46]. Still, the effect of EOs on preformed biofilms has been reported by several researches. For example, Sadekuzzamana et al. [47] found that thyme and tea tree EOs were effective in reducing *L. monocytogenes* biofilms. Sandasi et al. [25] showed that the inhibition of a preformed biofilm of *L. monocytogenes* was inhibited by at least 50% using *Rosmarinus officinalis*, *M. piperita*, and *Melaleuca alternifolia* extracts. Leonard et al. [48] found that *Mentha spicata* and *Syzigium aromaticum* EOs, as well as pure citral and nerol, were good candidates to inhibit *L. monocytogenes* biofilms.

The activity of the EOs used in this work has been investigated by other researchers against other biofilm-forming pathogens. EOC inhibited the biofilm formation of Acinetobacter [49] completely. De Oliveira et al. [50] found that 2% (v/v) of *C. cassia* was capable of reducing a 48 h preformed biofilm to below the detection limit. Garlic extract inhibited biofilm formation of *Streptococcus pneumoniae* (88.51%), *Bacillus cereus* (88.61%), and *Staph. aureus* (52.8%) [51]. Finally, a concentration of 50 mg mL^−1^ of an extract of red onion showed a reduction rate of 59.5% and 61.5% against *Staph. aureus* and *Ps. aeruginosa* biofilms, respectively [52]. EOs, due to the volatile nature of several of their compounds, can diffuse through the matrix of the biofilm, better than other antimicrobial agents, which could explain, at least in part, their antibiofilm performance [53].

Preformed biofilms were less inhibited at longer incubation times than at shorter ones at relatively low doses of EOs (0.5 and 1 MIC). Probably, at these low doses, biofilm cells can even adapt and start growth, but at higher doses (2 MIC), the inhibition persists during all the contact time with the EO. In fact, the decrease in effectiveness along with incubation could be explained by the starvation of the bacterial cell to nutrients and the need for an oxygen supply, which would lead to reduced metabolic activity and is accompanied by an increase in resistance [26]. In other words, antimicrobials are efficient only against metabolically active cells. Since the cells located in the upper biofilm layers consume all available oxygen and grow aerobically, an anaerobic micro-niche is developed underneath the aerobic layer. Oxygen- and nutrient-depleted regions are found at the bottom layers of the biofilm structure and, under these circumstances, most of the sessile cells are metabolically inactive, which would explain the increased resistance or death [25,54]. Bridier et al. [55] reported that the growth alteration might modify membrane composition and the expression of defense mechanisms, which could also be reasons for the decrease in antimicrobial effectiveness.

These findings seem promising for the food industry as *L. monocytogenes* is able to survive even in severe conditions. The bioactive compounds identified could be used within commercial sprays that could be vaporized to enhance their access to the biological targets and to prevent microbial adhesion to surfaces in the food industry. In addition, research into testing new EOs and antimicrobial combinations with different spectra and mode of action, looking for synergisms, may provide new ways of combating biofilms.

## 5. Conclusions

This investigation revealed that the EOs of garlic, onion, and cinnamon have a great antibiofilm effect against *Listeria monocytogenes*. All three EOs were able to inhibit initial cell attachment fully. Preformed biofilms were more resistant, and the inhibition rate was lower.

## Figures and Tables

**Table 1:** Chemical composition of essential oils of garlic (EOG), onion (EOO), and cinnamon (EOC).

		Concentration (% Peak Area)		
No.	Compound	EOG	EOO	EOC
1	sulfide allyl mehtyl	1.41	-	-
2	1,2-dithiolane	0.30	-	-
3	diallyl sulfide	20.89	-	-
4	allyl methyl disulfide	1.12	-	-
5	diallyl disulfide	22.74	-	-
6	trisulfide, methyl 2-propenyl	2.94	-	-
7	4-mehtyl-1,2,3-trithiolane	0.58	-	-
8	3,vinil-1,2-dithiacyclohex-5-nne	0.52	-	-
9	diallyl trisulfide	25.13	-	-
10	1-allyl-3-propyl trisulfane	0.27	-	-
11	5-methyl,1,2,3,4-tetrathiane	0.43	-	-
12	tetrasulfide, di-2-propenyl	13.54	-	-
13	6,htyl-4,5,7-trithia-2,8-decadiene	0.54	-	-
14	1,allyl-3-(2 allylthio)propyl)trisulfane	0.93	-	-
15	dimethyl disulfide	-	0.07	-
16	ropyl ydrodisulfide	-	0.09	-
17	propyl sulfide	-	0.33	-
18	disulfide, mehtyl propyl	-	5.11	-
19	dimehtyl trisulfide	-	0.25	-
20	dipropyl disulfide	-	31.11	-
21	trisulfide, mehtyl propyl	-	6.69	-
22	trisulfide, dipropyl	-	35.46	-
23	tetrasulfide, dipropyl	-	17.65	-
24	benzaldehyde	-	-	0.97
25	benzaldehyde, 2-hydroxy	-	-	0.21
26	phenyl ethyl alcohol	-	-	0.93
27	benzenepropanal	-	-	0.49
28	benzofuran, 2-mehtyl-	-	-	0.17
29	octanoic acid, ethyl ester	-	-	0.05
30	(Z)-cinnamaldehyde	-	-	0.37
31	3-phenylpropanol	-	-	0.07
32	benzaldehyde,2-methoxy-	-	-	0.22
33	acetic acid, 2-phenylethyl ester	-	-	0.08
34	E-cinnamaldehyde	-	-	76.54
35	cinnamyl alcohol	-	-	0.30
36	copaene			0.30
37	coumarin	-	-	2.59
38	acetic acid, cinnamyl eeter	-	-	4.21
39	(Z)-2-mehtoxy cinnamaldehyde	-	-	10.30

**Table 2:** Minimal inhibitory concentration (MIC) (mg mL^−1^) and zones of growth inhibition (mm) of garlic, cinnamon, and onion essential oils on *L. monocytogenes*. Zones of growth inhibition values are represented as mean ± standard deviation (including the disc diameter, 6 mm).

Essential Oil	MIC	Inhibition Zone
Garlic	0.100	31.0 ± 1.7 ^b^
Cinnamon	0.100	12.3 ± 0.5 ^a^
Onion	0.025	37.8 ± 0.4 ^c^

^a–c^ Different superscript letters indicate significant differences (*p* < 0.05).
